# Hidden biodiversity in Neotropical streams: DNA barcoding uncovers high endemicity of freshwater macroinvertebrates at small spatial scales

**DOI:** 10.1371/journal.pone.0231683

**Published:** 2020-08-07

**Authors:** Luis F. De León, Aydeé Cornejo, Ronnie G. Gavilán, Celestino Aguilar

**Affiliations:** 1 Department of Biology, University of Massachusetts Boston, Boston, MA, United States of America; 2 Centro de Biodiversidad y Descubrimiento de Drogas, Instituto de Investigaciones Científicas y Servicios de Alta Tecnología (INDICASAT AIP), Panamá, República de Panamá; 3 Smithsonian Tropical Research Institute, Balboa, Panamá; 4 Instituto Conmemorativo Gorgas de Estudios de la Salud, Panamá, República de Panamá; 5 Centro Nacional de Salud Pública, Instituto Nacional de Salud, Lima, Perú; 6 Escuela Profesional de Medicina Humana, Universidad Privada San Juan Bautista, Lima, Perú; King's College London, UNITED KINGDOM

## Abstract

Aquatic macroinvertebrates play a crucial role in freshwater ecosystems, but their diversity remains poorly known, particularly in the tropics. This “taxonomic void” limits our understanding of biodiversity patterns and processes in freshwater ecosystems, and the scale at which they operate. We used DNA barcoding to estimate lineage diversity (and the diversity of unique haplotypes) in 224 specimens of freshwater macroinvertebrates at a small spatial scale within the Panama Canal Watershed (PCW). In addition, we compiled available barcoding data to assess macroinvertebrate diversity at a broader spatial scale spanning the Isthmus of Panama. Consistently across two species delimitation algorithms (i.e., ABGD and GMYC), we found high lineage diversity within the PCW, with ~ 100–106 molecular operational taxonomic units (MOTUs) across 168 unique haplotypes. We also found a high lineage diversity along the Isthmus of Panama, but this diversity peaked within the PCW. However, our rarefaction/extrapolation approach showed that this diversity remains under-sampled. As expected, these results indicate that the diversity of Neotropical freshwater macroinvertebrates is higher than previously thought, with the possibility of high endemicity even at narrow spatial scales. Consistent with previous work on aquatic insects and other freshwater taxa in this region, geographic isolation is likely a main factor shaping these patterns of diversity. However, other factors such as habitat variability and perhaps local adaptation might be reshaping these patterns of diversity at a local scale. Although further research is needed to better understand the processes driving diversification in freshwater macroinvertebrates, we suggest that Neotropical streams hold a high proportion of hidden biodiversity. Understanding this diversity is crucial in the face of increasing human disturbance.

## Introduction

Aquatic macroinvertebrates are a fundamental component of freshwater environments. They mediate important processes such as food web dynamics, energy flow, and nutrient cycling, and therefore play a central role in sustaining the biodiversity and functioning of freshwater ecosystems [1–3]. However, the diversity of Neotropical freshwater macroinvertebrates remains poorly described, and even less is known about the processes that drive their diversity, and the scale at which they operate [4]. For instance, despite considerable efforts by local taxonomists (e.g., [5–8], most of the published literature use genus and family as a standard taxonomic unit for Neotropical macroinvertebrates (e.g., [9–14]⁠. This is partially due to the complexity of these communities, which are often composed of multiple life-stages existing at the interface between the terrestrial and aquatic environment [15,16]⁠. Another limitation is the low efficiency of traditional morphological methods, which are generally time-consuming, and highly variable in the quality of identification across taxa and experts.

This “taxonomic void” has important consequences for our general understanding of biodiversity patterns and processes, both in Neotropical environments and globally. For example, species diversity is generally expected to increase at lower latitudes [17,18]⁠, but no consensus has been reached for macroinvertebrates, given the current lack of taxonomic knowledge [19–22]. Within the Neotropics, our current understanding of the drivers of species diversity in benthic macroinvertebrates is also limited [23–25].

Similar to other freshwater taxa (e.g., [26,27], spatial isolation is likely a major factor driving diversification in macroinvertebrates, but few studies have tested this expectation [25,28,29]. In particular, Múrria et al. [25] found a high frequency of unique haplotypes associated with the geographical distance across watersheds in Panama. While confirmatory, these findings are not surprising, given the large geographic distance among the watersheds included in Múrria et al. [25]. However, patterns of haplotype (or lineage) diversity at smaller scales (e.g., among streams within watersheds), where dispersal and gene flow might be less restricted, have received less attention. We use DNA-barcoding to assess patterns of lineage diversity (and the diversity of unique haplotypes) in freshwater macroinvertebrates in four streams within the Panama Canal Watershed (PCW). In addition, we compiled available barcoding data [25] to contrast macroinvertebrate diversity at a broader spatial scale, among eight streams along the Isthmus of Panama.

Assessing the patterns and drivers of macroinvertebrate diversity at different scales is particularly relevant, given the increasing rate of environmental degradation in Neotropical regions [30–33]. This includes alterations such as introduction of alien species [34,35], the development of megaprojects [36], habitat degradation, water pollution, and climate change [30,33,37,38]⁠. As a consequence, a large portion of this biodiversity risks being lost before discovery.

## Material and methods

### Study sites and sample processing

**Fig 1:**
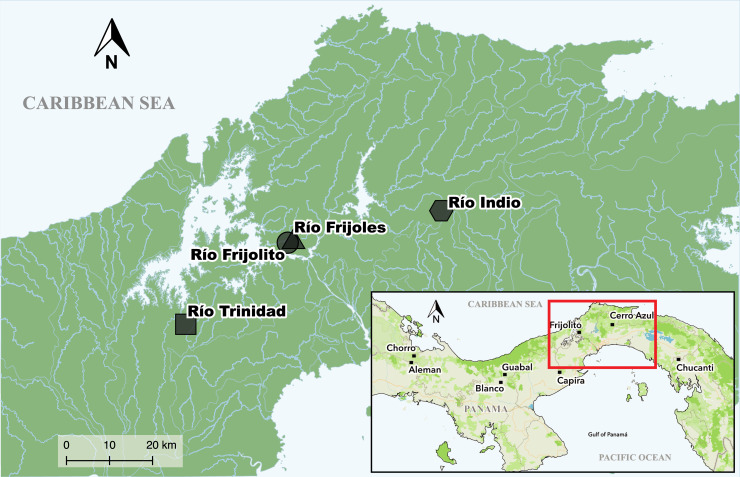
Sampling sites of macroinvertebrates in the Panama Canal Watershed. Names on the inset map indicate the sites previously sampled by Múrria et al. [25].

Samples were collected from four streams within the PCW (Frijolito, Frijoles, Trinidad, and Indio) between April and May of 2013 (Fig 1). Frijolito (09º08'57.9'' N, 79º43'53.2'' W) and Frijoles (09º09'08.2'' N, 79º44'05.3'' W) are typical Neotropical streams separated by approximately 300 m and located inside Soberanía National Park. These streams are surrounded by dense secondary forest and present low levels of disturbance. Río Trinidad (8º58'28.50'' N, 79º57'23.9'' W) is located approximately 30 km West Río Frijoles in an agricultural landscape dominated by pasture, but it has abundant riparian vegetation. Río Indio (09º12'04.1'' N, 079º24'20.4'' W), located 35 Km east of Frijoles, is intermediately disturbed and is surrounded by secondary forest with dense riparian vegetation. At each site, we haphazardly collected aquatic macroinvertebrates using standard kick-netting from the two dominant habitats types (riffles and pools). The sampling effort was approximately two hours at each site. All samples were sorted in the field and immediately preserved in 95% ethanol. Sampling permit was obtained from the Autoridad Nacional del Ambiente de Panamá (Permit No. SC/A-44-12).

In the laboratory, specimens were morphologically identified to the lowest possible taxonomic level (i.e., family or genus) using taxonomic keys for Neotropical macroinvertebrates [6,8,9,39]. However, given the low accuracy of morphological identification, and the fact that less than 50% of the individuals were successfully identified to species level using our barcoding data (see results), we focused our analyses and discussion on lineage rather than morphological diversity. Representative specimens have been deposited in the invertebrate collection at Colección Zoológica Dr. Eustorgio Méndez (CoZEM) at Instituto Conmemorativo Gorgas de Estudio de la Salud in Panama City (Voucher numbers: B001 –TR020).

### DNA sequencing

Tissue samples were obtained from the hind leg or part of the body of each specimen, and total DNA was extracted by using the DNeasy Blood & Tissue kit (Qiagen, CA, USA), according to the manufacturer’s instructions. A standard sequencing protocol [40] was used to amplify the full-length 658 base pair (bp) of the COI barcode region using the following primers sets: LCO1490/HCO2198 [41] and LepF1/LepR1 [42]. All PCR products were verified on a 1% agarose gel, and purified with EXO-SAP-IT (USB Corp., Cleveland, Ohio, U.S.A.). The protocol included adding 1 μl of 1 U/μl Shrimp Alkaline Phosphatase (SAP), 0.5 μl of 20 U/μl Exonuclease I (EXO), and 5 μl of amplified product, and then incubating as indicated in the manufacturer's protocol [43]. This product was sequencing using an Applied Biosystems Genetic Analyzer (ABI 3130xl, Applied Biosystems, Carlsbad, California). Sequences were aligned in Geneious V7.03 [44], using the MAFFT 7.313 [45] tool and the L-INS-i algorithm. Sequence alignments were also inspected by eye in Geneious to confirm overall sequence quality. We did not find gaps or stop codons in any of the sequences. Project sequences, together with the information on collected specimens, are available on the Barcode of Life Data System (BOLD systems, under project code INVPA: http://www.boldsystems.org/). Project sequences are also available in GenBank (accession numbers: KX039451-KX039650, KU980966-KU981004).

### Data analysis

To confirm morphological identification for our sequenced specimens, we performed BLAST searches for publicly available sequences in GenBank. We created a final dataset comprising unique haplotypes from our study and including three COI sequences retrieved from Genbank that were used as an outgroup. The retrieved sequences were *Thermobia domestica* (GenBank NC006080), *Atelura formicaria* (GenBank NC01119), and *Tricholepidion gertschi* (GenBank NC005437).

We estimated phylogenetic relationships among taxa using maximum likelihood (ML) searches in IQ-TREE v 1.6 [46] and Bayesian inference (BI) in BEAST v 2.4.6 [47] as implemented on the CIPRES Science Gateway [48]. The best-fit model of nucleotide substitution for the dataset, selected using jModelTest 2.0 [49] based on the Bayesian Information Criterion, was GTR+I+G. To determine node support for the IQ-TREE we used 10000 ultrafast bootstraps [50] and 1000 Shimodaira-Hasegawa-like approximate likelihood ratio test replicates [51]. BI analysis was executed with an uncorrelated lognormal relaxed clock and coalescent prior, with the default settings of BEAUti for the remaining parameters. We performed two runs of 2.0×10^7^ generations, and sampled trees every 5000 generations. Trace logs and species trees for the two runs were combined using LogCombiner v 2.4.8 [52]. We used Tracer v. 1.6 [53] to ensure that effective sample size (ESS) values for all parameters were above 200 and to determine the burn‐in. Finally, output trees were summarized as maximum clade credibility (MCC) trees using mean node heights after discarding 25% of generations as burn-in using TreeAnnotator v1.8.4 [54].

We then assessed the diversity of “molecular species” by estimating molecular operational units (MOTUs; [55] using the software MEGA 7.0 [56] and the BOLD analyses tools [57]. Sequence divergence was estimated using the Kimura-2-Parameter (K2P) model with 1000 bootstrap estimates in MEGA7. This a standard model that has been extensively used in barcoding studies [58,59]. The Barcode Index Number (BIN) system [57] was used as delimitation criterion for the assignment of MOTUs across the full dataset. This method uses a 2.2% in sequence divergence cut-off, but updates this value according to the distribution of divergence among sequences in the dataset. Recent studies suggest that using a single divergence cut-off may not be appropriate for every organism (reviewed in [60], such as in the case of diverse non-tropical chironomids [60,61]. But, given that our sample of this taxon was small, we assumed a single cut-off value for the entire dataset. However, future work should evaluate the optimal level of genetic divergence to delimit biological species in Neotropical macroinvertebrates.

In addition, given recent concerns with the use of the K2P model for species delimitation (e.g., [62], we applied two additional single-locus analyses to confirm lineage diversity: the Bayesian General Mixed Yule Coalescent model (GMYC; [63] and the Automatic Barcode Gap Discovery (ABGD; [64]. The GMYC approach uses branch lengths to determine the transition from intraspecific to interspecific relationships [63]. The ABGD algorithm allows to sort DNA sequences into “hypothetical species” based on the gaps in the distribution of intra- and inter-specific genetic divergence in a given sample [64]. Although the two approaches differ in their properties (i.e., tree branch length vs. distribution gaps), we used them to confirm the patterns of species delimitation.

To perform GMYC tree-based analyses, we used the ultrametric trees previously generated with BEAST. GMYC was performed using the single threshold parameter at the GMYC webserver (https://species.h-its.org/gmyc/). ABGD was carried out using the online version of ABGD software [64]; https://bioinfo.mnhn.fr/abi/public/abgd/abgdweb.html). Default settings were used, however, distance matrices based on K2P distance calculated in MEGA7 were used as input. All analyses were run using a relative barcoding gap width (X value) set to 1.0. Only the recursive results were used because they allowed for different gap thresholds among taxa.

To compare patterns of spatial variation in genetic diversity (MOTUs), we quantified the number of shared species among sampling sites. We also estimated Fisher's alpha index of diversity and Whittaker’s measure of β- beta diversity. Given that these analyses might be affected by variation in sampling size, we also used rarefaction and extrapolation methods [65,66] as implemented in the R package iNEXT [67]. This method allows for comparisons between sites while controlling for differences in abundance and sampling effort. For these analyses, we fit curves for the first three Hill numbers: species richness (q = 0), the exponential of Shannon entropy (“Shannon diversity”, q = 1), and the inverse Simpson concentration (“Simpson diversity”, q = 2), using individual-based abundance data. Given the limitation in sample size, we did attempt to make statistical inferences regarding differences in diversity among sites.

Finally, to assess lineage diversity and the diversity of unique haplotypes across watersheds spanning the Isthmus of Panama, we collected haplotype information (426 haplotypes: GenBank accession numbers KR134410-KR134835) from a previous study in this region [25]. After adding these sequences to our dataset, we generated multiple sequence alignments with MAFFT 7.313 [45] using the L-INS-i algorithm in Geneious V7.03. Then, we trimmed the sequences to the same fragment size and compared the previously reported haplotypes with the ones encountered in our dataset. To exclude redundancies prior to phylogenetic analyses, we applied DAMBE v. 6.4.11 [68] to identify and remove duplicate haplotypes from our dataset. In total, we found a total of 15 duplicate haplotypes between the two datasets. The combined dataset contained 12 sites (8 from Múrria et al. [25], and 4 from the present study). One site (Frijolito) was sampled during both studies, but we analyzed them separately to preserve independence between the two studies. We then applied the same rarefaction/extrapolation approach described above to generate rarefaction curves as a function of the number of individuals sampled. Although the collection method was similar (i.e., both studies used kick-nets during a given amount of time), the two studies may not be directly comparable due to differences in sampling effort and the overall study objective. Therefore, we did not attempt to make statistical inferences about the relative abundance of macroinvertebrates among sites or between studies. Instead, we only focused on compiling the total number of unique haplotypes or molecular species that are currently known within each site in this region. In addition, our ultimate goal was to explore the overall molecular endemicity of aquatic macroinvertebrates, rather than providing precise estimates of species diversity in this region. Thus, substantially more research that includes more data, sites, and replication is needed to confirm if current patterns hold across the entire region.

## Results

We collected approximately 300 specimens across the four sites; however, our analysis focused on the 224 individuals that were successfully barcoded (Table 1 and S1 Table). We were able to identify nearly 70% of individuals to genus level using the morphological approach, but species-level identification was only possible for 56 individuals (25%). Some of the most numerous taxa across sites included Leptophlebiidae (11.2% of individuals), Libellulidae (11.2%), Naucoridae (6.7%), Notonectidae (6.3%), Chironomidae (5.4%), Gerridae (4.9%), Hydropsychidae (4.0%), Perlidae (3.6%), and Baetidae (3.6%).

**Table 1:** Macroinvertebrate lineage diversity in the Panama Canal Watershed.

Site	Ind.	Hap.	GMYC	ABGD
Trinidad (TRI)	45	33	22	21
Frijolito (FTO)	65	46	27	26
Frijoles (FES)	88	72	52	50
Indio (IND)	26	24	22	21
All	224	168	106	100

The data show the number of successfully barcoded individuals (Ind.), cox1 haplotypes (Hap.), and molecular species (based on GMYC and ABGD) collected at four sites.

The 224 COI sequences revealed a total of 168 haplotypes (S1 Table). After comparing these haplotypes with molecular data from Múrria et al [25], we found 153 (~91%) unique haplotypes from the Panama Canal Watershed. In Frijolito, the site sampled by the two studies, we found a total of 46 haplotypes, 45 of which were unique to our study.

Our final COI dataset consisted of 171 terminals, including the 168 new barcode sequences, and 3 outgroup sequences retrieved from GenBank. The final aligned and pruned dataset contained 620 aligned positions, including gaps, with 371 variable sites, of which 359 were parsimony-informative (~96% of variable positions). As expected, we observed a hierarchical increase in the mean K2P genetic divergence with increasing taxonomic levels from within a species 0.38% (SE = 0.002), to within family 9.92% (SE = 0.01), to within order 19.75% (SE = 0.01). However, we were not able to identify our specimens to species level from our BLAST search, given that only around 50% of our sequences matched existing data in the public databases, and most of these matches corresponded to genus and family level only. Both ML and BI inference trees for all specimens showed well-defined clades at the level of order and family, with some differences in the topology, but overall support was higher for the BI tree, which we used to represent the number of molecular species (S1 Fig and S2 Fig).

Our species delimitation analyses yielded variable, but relatively high numbers of species. Specifically, GMYC detected 106 MOTUs (95% confidence intervals: 104–109), whereas ABGD found a total of 100 MOTUs (S1 Fig). These ABGD results were confirmed independently of the chosen model (Jukes-Cantor and Kimura) and were unaffected by changes of prior limits for intraspecific variation and threshold.

**Fig 2:**
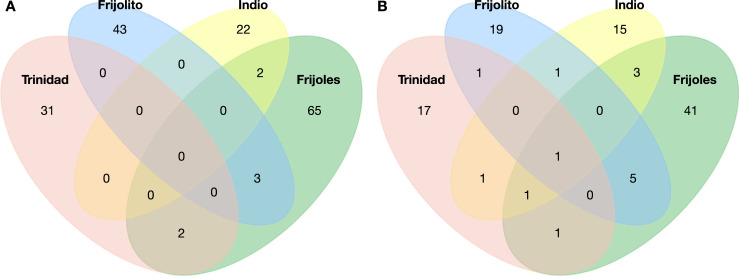
Distribution of molecular diversity in freshwater macroinvertebrates among streams within Panama Canal Watershed. Venn diagrams show the number of shared and unique haplotypes (A) and MOTUs (B) across four streams: Trinidad (pink), Frijolito (blue), Indio (yellow), and Frijoles (green).

**Fig 3:**
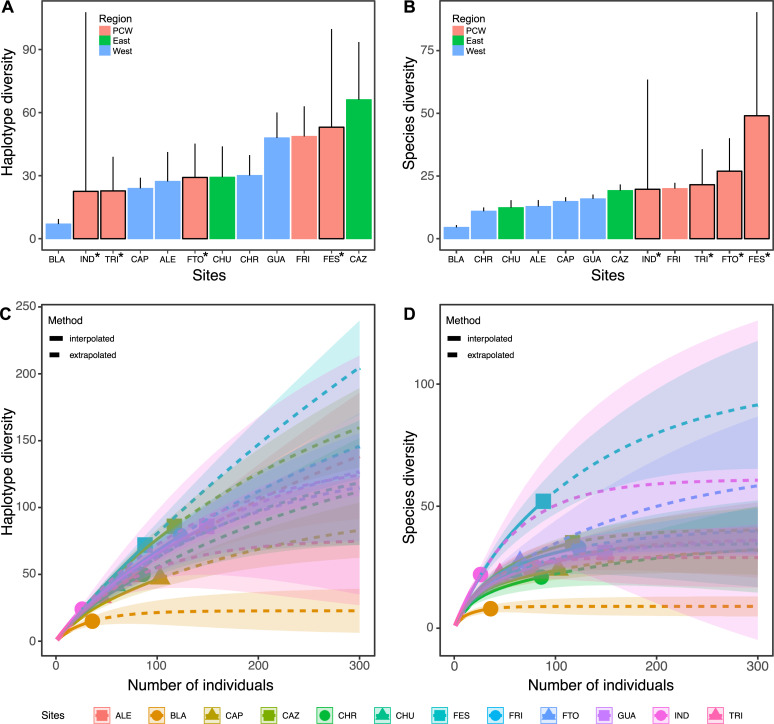
Molecular diversity in freshwater macroinvertebrates along the Isthmus of Panama. Panels show Simpson's diversity index for both haplotype (A) and MOTUs (B), as well as rarefaction and extrapolation curves for both haplotypes (C) and MOTUs (D) diversity at each site. Sites are Alemán (ALE), Chorro (CHO), Blanco (BLA), Guabal (GUA), Capira (CAP), Frijolito (FRI), Cerro Azul (CAZ), and Chucantí (CHR) from Múrria et al. 2015; and Trinidad (TRI), Frijolito (FTO), Frijoles (FES) and Indio (IND) from present study (indicated with asterisks). Regions are indicated as East, West and the Panama Canal Watershed (PCW).

When looking at spatial patterns of diversity, we observed some overlap in the number of shared MOTUs as well as a considerable number of unique haplotypes in each river: Frijoles (65), Frijolito (43), Trinidad (31) and Indio (22) (Fig 2). This pattern was also supported by the Fisher’s alpha diversity index, which showed variation in molecular species among sites: Frijoles 53.40, Frijolito 22.80, Trinidad 17.01, and Indio 67.63. Whittaker’s index of β diversity also showed high species turn-over across sites (0.87). Similarly, our rarefaction/extrapolation analyses showed variation in species richness across sites: Frijoles (52), Frijolito (27), Trinidad (22) and Indio (22). However, the most striking pattern was a lack of saturation in the accumulation curves for most of the sites (Fig 3), and this pattern was consistent across the first three Hill numbers (S3 Fig). Similar results were found when looking at diversity Hill across the Isthmus of Panama using the compiled barcoding dataset (Fig 3). In particular, we observed substantial diversity of both MOTUs and unique haplotypes across sites, but the accumulation curves did not reach saturation for most of the sites (Fig 3). In addition, both molecular species and haplotype diversity tended to increase at sites within the PCW, in contrast to sites located in the eastern and western portion of the country (Fig 3). Data on assignment and diversity of MOTUs across study sites are available in the supplementary material (S1 Table).

## Discussion

Using DNA-barcoding, we examined the diversity of freshwater macroinvertebrates at a small spatial scale, among four streams within the Panama Canal watershed (PCW). We also compiled existing barcoding data [25] to contrast macroinvertebrate diversity at a broader spatial scale, across eight streams along the Isthmus of Panama. Overall, we found high lineage diversity across sites within the PCW (Table 1; S1 Fig), and a large portion of these lineages appear to be unique to each site (Fig 2). In addition, our rarefaction/extrapolation approach showed that this diversity is still under-sampled across sites both within the PCW and along the Isthmus of Panama (Fig 3).

These findings confirm that the diversity of freshwater macroinvertebrates in Neotropical environments is largely under-studied [33,69,70], and could be much higher than previously thought. In particular, these findings highlight the fact that there is limited published research using genetic methods to study macroinvertebrate diversity in this region. This was reflected by one of our sites (Frijolito), which despite being sampled by a previous barcoding study [25], still showed a substantial number of novel haplotypes. In addition, the fact that only a small number of our specimens matched available sequences in public databases further highlights the potential for biodiversity discovery in Neotropical freshwater environments. This seems particularly relevant for taxa such as Hydropsychidae, Gerridae, Chironomidae, Leptophlebiidae, Libellulidae and Notonectidae, which showed high lineage/haplotype diversity across sites (S1 Fig; S1 Table). Some of these taxa also showed high haplotype diversity in a previous molecular study across Panama [25], and are thought to hold a high number of undescribed species in the Central American Isthmus [6,69]. Unfortunately, our analysis is limited by relatively small sample size, particularly at one of our sites (Río Indio), where we were only able to sequence 26 specimens. In addition, the fact that our rarefaction/extrapolation analysis showed a lack of saturation for most of the sites indicates that substantially more research is needed in this region. Overall, however, our results are in line with recent work showing high haplotype endemicity among isolated watersheds across the Isthmus of Panama [25]. In fact, despite low sample size, we found at least 153 (~91%) novel haplotypes within the PCW. Thus, we expand on this previous work by highlighting the possibility that endemicity of Neotropical macroinvertebrates can be substantial even within a single watershed.

Typically, diversification in freshwater organisms is marked by a strong geographic signature, where genetic divergence is facilitated by spatial isolation among populations [26,27]. However, the contribution of geographic isolation to the diversification of Neotropical freshwater macroinvertebrates has received little attention to date [25]. In addition, the fact that most macroinvertebrates are semiaquatic, and are likely to disperse during the adult stages [15,16] could limit genetic isolation among nearby stream communities. Yet, the possibility of high endemicity even within a single watershed suggests that spatial isolation, habitat variability, and perhaps, local adaptation are important drivers of macroinvertebrate diversity. Another interesting finding was that macroinvertebrate diversity appeared to increase at sites located in Central Panama, specifically within the PCW (e.g., Frijoles, Frijolito, Trinidad). However, additional research is needed to confirm this pattern and to explore the underlying drivers. We encourage the application of more efficient tools such as DNA metabarcoding to facilitate this endeavor.

Our finding of high endemicity at a small geographic scale is also relevant in the face of increasing anthropogenic disturbances [31,32,71–73]. Specifically, it suggests that small-scale local disturbances could have drastic consequences for the maintenance of a unique freshwater biodiversity–but this diversity is still unknown. We therefore predict that the current rate of species loss in freshwater ecosystems might be surpassing the rate of species discovery in Neotropical environments. Overall, however, further work is clearly needed to disentangle the contribution of other factors such as genetic drift, local adaptation, and environmental disturbance to persistence and diversification of Neotropical freshwater macroinvertebrates.

Taken together, our results confirm the expectation that the diversity of Neotropical macroinvertebrates remains under-studied. They also indicate that uncovering this hidden diversity is crucial to our understanding of the local and regional processes that shape biodiversity in Neotropical freshwater environments.

## Supporting information

S1 Fig Molecular diversity in freshwater macroinvertebrates from Central Panama. The Bayesian inference tree shows species delimitation analyses based on generalized mixed Yule coalescent (GMYC) and the automatic barcode gap discovery (ABGD). Black and grey blocks represent putative molecular species, with taxa sharing the same block corresponding to similar species. The numbers next to the nodes represent Bayesian posterior probability values.
(PDF)

S2 Fig Rarefaction and extrapolation curves for molecular diversity (MOTUs) at each site. Number at the top represent fit curves for the first three Hill numbers: species richness (q = 0), the exponential of Shannon entropy (“Shannon diversity”, q = 1), and the inverse Simpson concentration (“Simpson diversity”, q = 2), using individual-based abundance data. Sites are: Alemán (ALE), Chorro (CHO), Blanco (BLA), Guabal (GUA), Capira (CAP), Frijolito (FRI), Cerro Azul (CAZ), and Chucantí (CHR) from Múrria et al. 2015; and Trinidad (TRI), Frijolito (FTO), Frijoles (FES) and Indio (IND) (from the present study).
(PDF)

S3 Fig Phylogenetic tree determined by the Maximum Likelihood (ML). Data represent *cox1* sequences obtained from 224 freshwater macroinvertebrates collected within the Panama Canal Watershed. The numbers on the branches show nodal support.
(PNG)

S1 Table Molecular and taxonomic diversity of freshwater macroinvertebrates within the Panama Canal Watershed. For each specimen, we show a taxonomic group (i.e., order, family, and genus), molecular species identity (MOTUs: based on ABGD and GMYC), Haplotype identity, Genbank accession number, and sampling site. Sites are Trinidad (TRI), Frijolito (FTO), Frijoles (FES) and Indio (IND).
(XLSX)
