# Supplementary figures and images for: Hidden biodiversity in Neotropical streams: DNA barcoding uncovers high endemicity of freshwater macroinvertebrates at small spatial scales
Source: PLoS One. 2020 Aug 7;15(8):e0231683. doi: 10.1371/journal.pone.0231683 (PMC7413512; doi:10.1371/journal.pone.0231683)

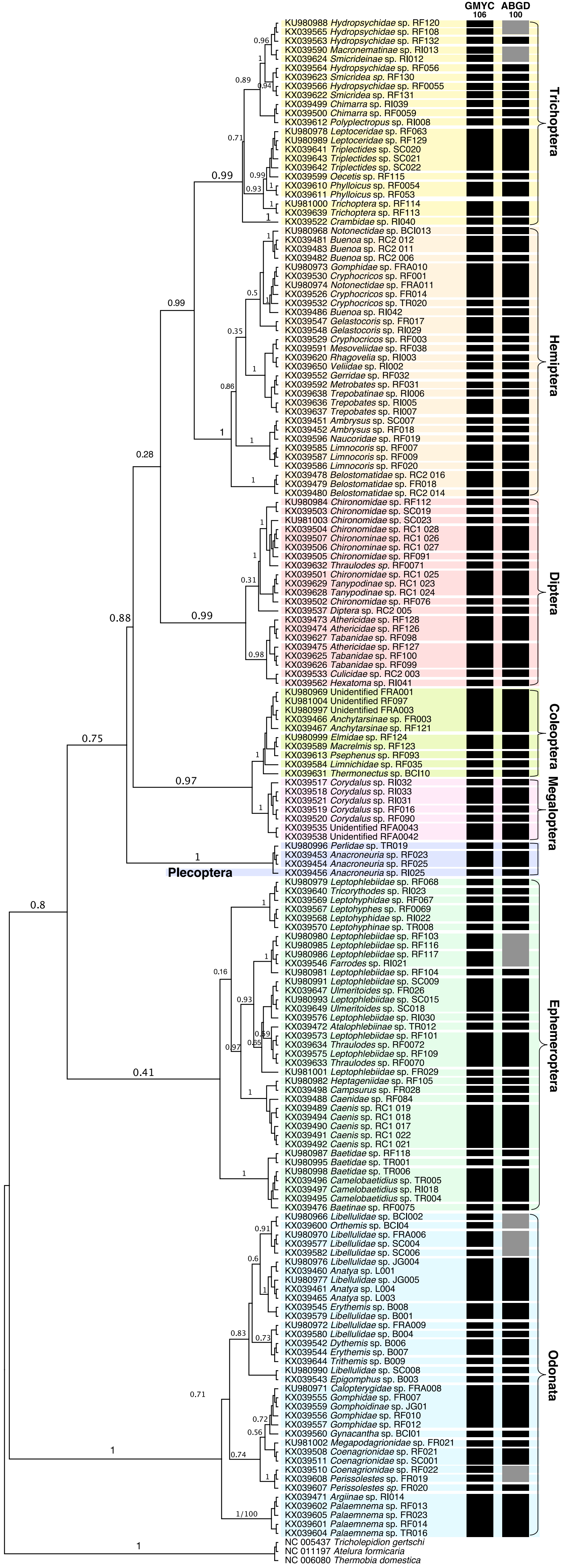

Supplement: S1 Fig — The Bayesian inference tree shows species delimitation analyses based on generalized mixed Yule coalescent (GMYC) and the automatic barcode gap discovery (ABGD). Black and grey blocks represent putative molecular species, with taxa sharing the same block corresponding to similar species. The numbers next to the nodes represent Bayesian posterior probability values. (PDF) [file pone.0231683.s001.pdf]

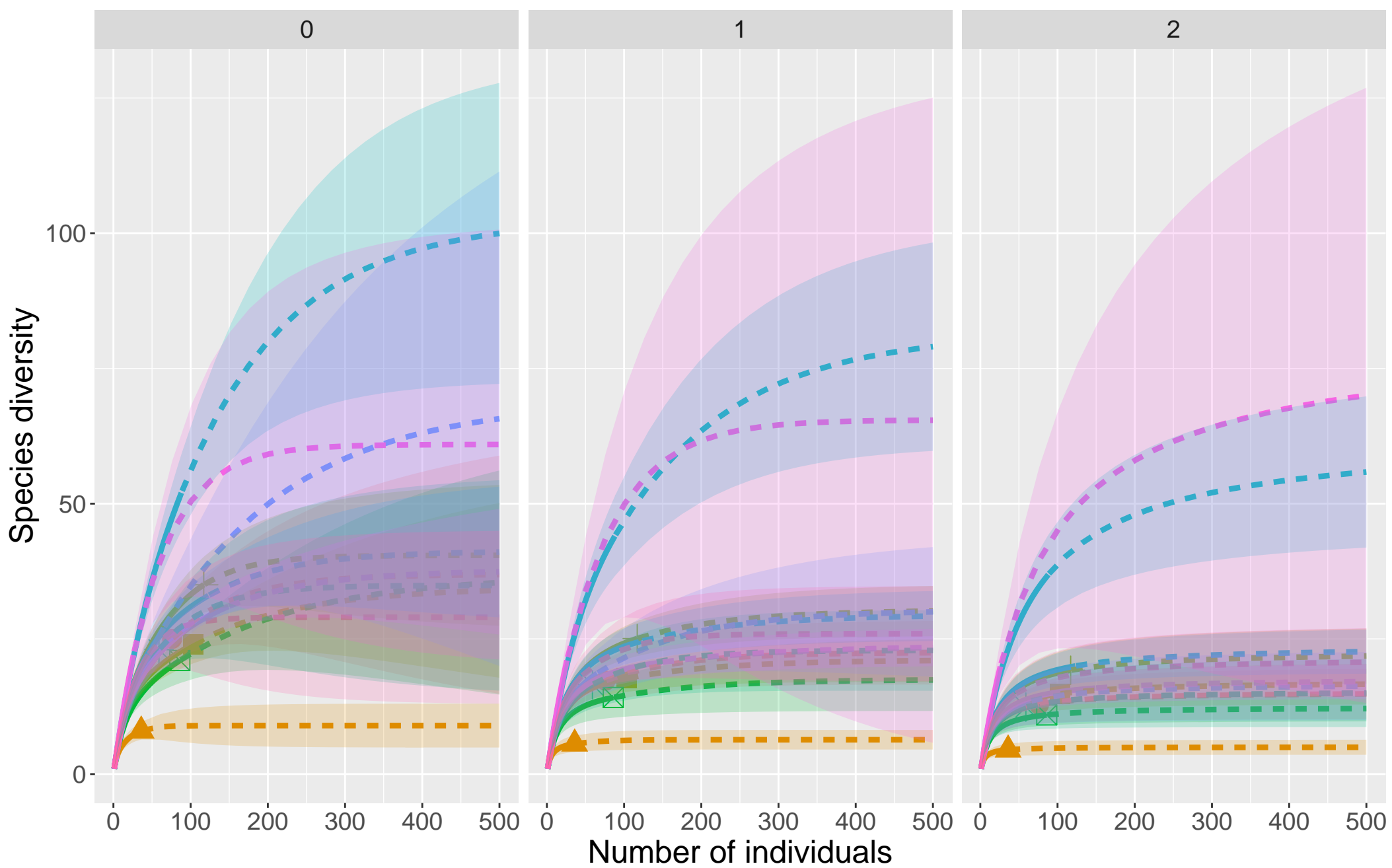

— interpolated    - - - extrapolated

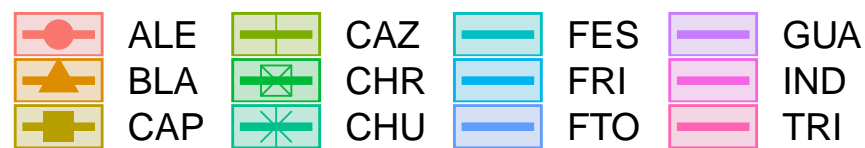

Supplement: S2 Fig — Number at the top represent fit curves for the first three Hill numbers: species richness (q = 0), the exponential of Shannon entropy (“Shannon diversity”, q = 1), and the inverse Simpson concentration (“Simpson diversity”, q = 2), using individual-based abundance data. Sites are: Alemán (ALE), Chorro (CHO), Blanco (BLA), Guabal (GUA), Capira (CAP), Frijolito (FRI), Cerro Azul (CAZ), and Chucantí (CHR) from Múrria et al. 2015; and Trinidad (TRI), Frijolito (FTO), Frijoles (FES) and Indio (IND) (from the present study). (PDF) [file pone.0231683.s002.pdf]

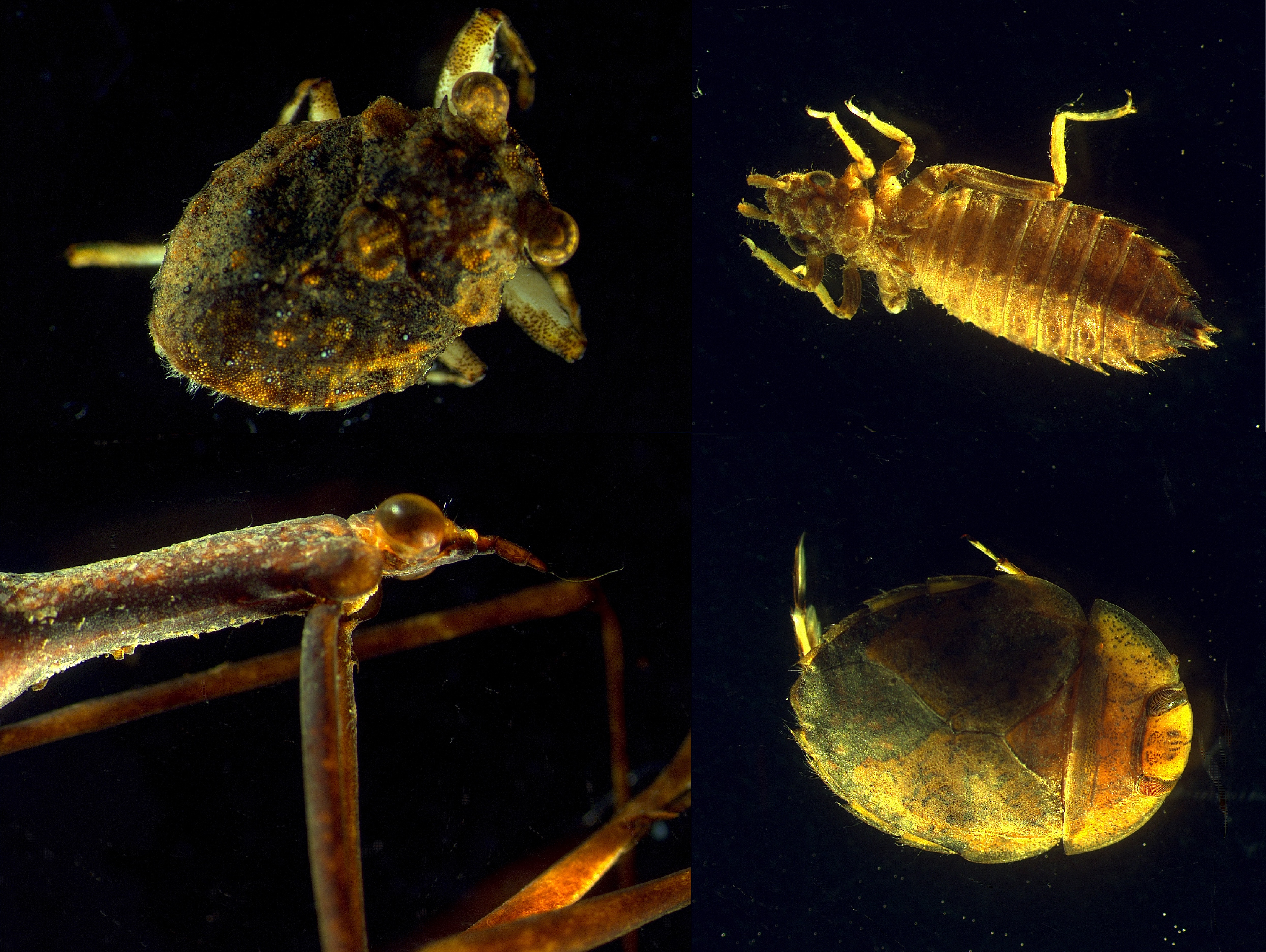

Supplement: S3 Fig — Data represent cox1 sequences obtained from 224 freshwater macroinvertebrates collected within the Panama Canal Watershed. The numbers on the branches show nodal support. (PNG) [file pone.0231683.s003.png]
